# Leprosy in blood donors

**DOI:** 10.1111/tmi.70007

**Published:** 2025-07-03

**Authors:** Erika Vanessa Oliveira Jorge, Angélica Rita Gobbo, Izabelle Laissa Viana Costa, Raquel Carvalho Bouth, Sâmela Miranda da Silva, Ana Caroline Cunha Messias, Josafá Gonçalves Barreto, Patrícia Fagundes da Costa, Pablo Diego do Carmo Pinto, Moises Batista da Silva, John Stewart Spencer, Maurício Koury Palmeira, Claudio Guedes Salgado

**Affiliations:** ^1^ Dermato‐Immunology Laboratory Federal University of Pará Marituba Pará Brazil; ^2^ Institute of Biological Sciences, Federal University of Pará Belém Pará Brazil; ^3^ Blood Bank of Pará State (Centro de Hematologia e Hemoterapia do Estado do Pará, HEMOPA) Belém Pará Brazil; ^4^ Laboratory of Spatial Epidemiology (LabEE), Federal University of Pará Castanhal Pará Brazil; ^5^ Human and Medical Genetics Laboratory Institute of Biological Sciences, Federal University of Pará Belém Pará Brazil; ^6^ School of Medicine, Institute of Medical Sciences, Federal University of Pará Belém Pará Brazil; ^7^ Department of Microbiology, Immunology and Pathology Colorado State University Fort Collins Colorado USA

**Keywords:** anti‐PGL‐I IgM serology, blood donor, leprosy, *Mycobacterium leprae*, PCR RLEP

## Abstract

**Objectives:**

We investigated the prevalence of anti‐phenolic glycolipid‐I (PGL‐I) IgM antibodies among temporarily unfit blood donors at the Pará State Blood Bank (HEMOPA), located in the Amazon region of northern Brazil. Using an arbitrary high cutoff for optical density (OD ≥0.750) in ELISA, a subset of donors was invited for clinical evaluation for leprosy.

**Methods:**

Temporarily unfit individuals were invited to participate, and blood samples were collected for anti‐PGL‐I IgM titration by ELISA. Donors with high OD values were referred for clinical examination, slit skin smear (SSS) bacilloscopy, and quantitative PCR (qPCR) targeting *M. leprae*‐specific repetitive element (RLEP) sequences from dermal scrapes of the earlobes and peripheral blood.

**Results:**

From an annual average of 2762 temporarily unfit donors (2019–2023), 500 (16.6%) were tested for anti‐PGL‐I IgM. Of these, 20/500 (4.0%) had high antibody titres, and 8/20 (40.0%) attended clinical evaluation, resulting in 5/8 (62.5%) newly diagnosed cases of leprosy. Among these, *Mycobacterium leprae* detection yielded positivity rates of 2/8 (25.0%) by SSS bacilloscopy, 3/7 (42.9%) by qPCR of dermal scrapes, and 2/8 (25.0%) by qPCR of peripheral blood.

**Conclusions:**

Using an anti‐PGL‐I IgM cutoff of OD ≥0.750, we identified a significant proportion of undiagnosed leprosy cases among temporarily unfit blood donors. These findings support the need for targeted leprosy screening in this population. Regardless of qPCR results, individuals with clinical signs of leprosy require appropriate treatment and assessment of their eligibility for blood donation.

## INTRODUCTION

Brazil ranked second globally for new leprosy cases in 2022, accounting for approximately 90% of all cases reported in the Americas [1]. Efforts to reduce the incidence of leprosy face significant challenges, primarily due to the difficulty in identifying people affected by leprosy, particularly during the early, oligosymptomatic, subclinical, or latent stages of the disease [2].

Leprosy is a chronic infectious disease caused by *Mycobacterium leprae* and *Mycobacterium lepromatosis*, a related species identified in other endemic regions such as Mexico and the Caribbean. The disease primarily affects peripheral nerves and the skin, but it may also involve other organs, including the eyes, testes, liver, and bone marrow. The infection leads to inflammation and progressive neurodegeneration, resulting in a spectrum of clinical manifestations ranging from tuberculoid (TT) to lepromatous leprosy (LL) forms. While TT and LL are immunologically stable, borderline forms (borderline tuberculoid [BT], borderline borderline, borderline lepromatous) may shift during disease progression [3]. Early diagnosis is critical to prevent progression to these more advanced clinical forms.

Transmission occurs mainly through respiratory secretions; however, there is historical evidence suggesting the possibility of blood‐borne transmission. Notably, in the 1960s, a case was reported in which twins developed leprosy following a blood transfusion from a patient with lepromatous leprosy. In the 1970s, viable *M. leprae* bacilli were isolated from the blood of leprosy patients and shown to replicate in mouse footpad models [4].

Blood donation programmes aim to ensure both donor and recipient safety through rigorous screening processes that maintain the quality of the blood supply while minimising unnecessary deferrals. In Brazil, individuals with systemic diseases that may compromise blood quality are permanently disqualified from donating blood [5]. Leprosy is classified as a condition that results in permanent ineligibility for blood donation.

Diagnosis of leprosy is primarily clinical, as conventional laboratory methods such as slit skin smear (SSS) microscopy for acid fast bacilli (AFB) and histopathological examination often have low sensitivity, particularly in early or latent infections. Serological assays detecting anti‐phenolic glycolipid‐I (PGL‐I) IgM antibodies and molecular techniques such as quantitative PCR (qPCR) targeting the RLEP sequence of *M. leprae* have demonstrated higher sensitivity and can aid in the diagnosis of early or subclinical cases, even when bacilloscopy is negative [2].

Recent studies emphasise the importance of detecting hidden leprosy cases within communities, highlighting the need for active surveillance in endemic regions. Moreover, the global decline in clinical expertise for leprosy diagnosis and the persistence of hidden endemicity in several countries underscore the urgency of strengthening transfusion safety protocols. This includes consideration of the potential risk of *M. leprae* transmission through blood donation, even in countries where leprosy is not currently considered endemic, such as the United States [6, 7].

## MATERIALS AND METHODS

### Study design

Temporarily unfit blood donors from the Pará State Blood Bank (HEMOPA), excluding those disqualified due to a prior leprosy diagnosis, were invited to participate and provided informed consent. Temporarily unfit donors were individuals deferred from donation due to transient conditions such as recent infections (e.g., hepatitis, syphilis), lipemic serum, or reactive serology for diseases unrelated to leprosy.

Participants were tested for anti‐PGL‐I IgM antibody titres against *M. leprae* using ELISA. Based on previous observations, individuals were stratified into three groups according to their ELISA optical density (OD) values: Group 1: OD <0.295 (considered seronegative; defined as the mean OD + 2 standard deviations of a healthy control population); Group 2: 0.295 ≤ OD <0.750 (seropositive); Group 3: OD ≥0.750 (high‐titre seropositive).

The threshold of 0.295 was established as the cutoff for seropositivity, while 0.750—approximately 2.5 times higher—was used to define high anti‐PGL‐I IgM titers. Individuals in Group 3 were invited for further clinical evaluation and laboratory testing at the Reference Unit in Sanitary Dermatology of Pará State, “Dr. Marcello Candia,” as high anti‐PGL‐I IgM titres (OD ≥0.750) are more likely to be associated with active *M. leprae* infection. Higher antibody levels have been linked to multibacillary forms of leprosy, reinforcing the importance of targeted clinical assessment in this subgroup.

### Serological screening of blood donors

Plasma samples were screened for anti‐PGL‐I IgM antibodies using an ELISA with the synthetic ND‐O‐BSA antigen (kindly provided by Dr. John Spencer, Colorado State University, USA), following previously established protocols [8]. Optical density was measured at 490 nm using an MRX Revelation 4.25 microplate reader (Dynex Technologies, Chantilly, VA, USA). All samples were tested in duplicate, and background‐corrected OD values were averaged. Samples with OD ≥0.295 were considered seropositive. Individuals with OD ≥0.750 were selected for clinical examination, SSS bacilloscopy and molecular testing.

### Identification of *Mycobacterium leprae*

Participants with high anti‐PGL‐I IgM titres who consented to further evaluation underwent SSS collection for microscopic detection of AFB and bacillary index determination using the Ridley logarithmic scale. Molecular detection was performed via qPCR targeting the RLEP repetitive element of *M. leprae* DNA extracted from dermal scrapes of both earlobes and peripheral blood samples. DNA extraction was carried out using the DNeasy Blood and Tissue Kit (Qiagen, Hilden, Germany), following the manufacturer's protocol. The RLEP region was amplified using the primer pair LP1 (5′‐TGCATGTCATGGCCTTGAGG‐3′) and LP2 (5′‐CACCGATACCAGCGGCAGAA‐3′), as previously described [2]. Amplification was performed using the 7500 Real‐Time PCR System (Applied Biosystems, Foster City, CA, USA), with a cycle threshold (Ct) cutoff of ≤45 cycles to define positivity.

## RESULTS

Between 2019 and 2023, the Pará State Blood Bank (HEMOPA) collected an average of 93,440 blood bags per year. Of these, an annual average of 2762 (2.96%) were classified as temporarily unfit for donation. Among 500 temporarily unfit donors tested for anti‐PGL‐I IgM antibodies, 339 (67.8%) were seronegative, 141 (28.2%) were seropositive, and 20 (4.0%) exhibited high anti‐PGL‐I IgM titres (OD ≥0.750).

**FIGURE 1:**
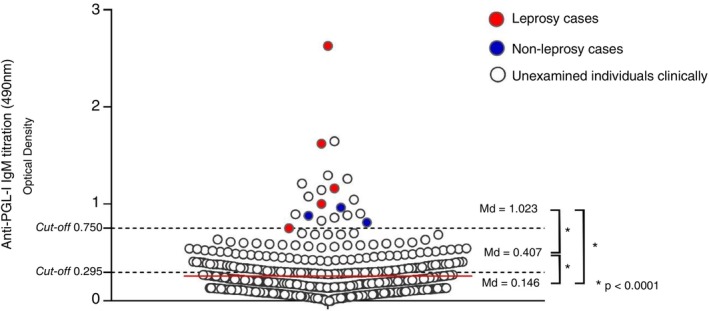
Profile of anti‐phenolic glycolipid‐I (PGL‐I) IgM antibody titration in temporarily unfit blood donors. A total of 500 participants were stratified into three groups based on anti‐PGL‐I IgM titration (optical density [OD] at 490 nm): (1) OD < 0.295; (2) 0.295 ≤ OD <0.750; and (3) OD ≥ 0.750. The three groups showed significantly different median OD values (*p < 0.0001, unpaired two‐tailed Mann–Whitney test). Group 3 (*n* = 20), with a median OD of 1.023, included individuals with high anti‐PGL‐I IgM titres. Of these, eight underwent clinical examination for leprosy: five newly diagnosed cases are highlighted in red, and three individuals not diagnosed are marked in blue. Uncoloured data points represent individuals who were not clinically examined.

The median OD values were as follows: 0.146 for the seronegative group, 0.407 for the seropositive group, and 1.023 for the high‐titre group. All three groups showed statistically significant differences in OD values (*p* < 0.0001) (Figure 1).

Of the 20 individuals with high anti‐PGL‐I IgM titres, 8 consented to clinical evaluation. Among them, 5/8 (62.5%) were newly diagnosed with multibacillary leprosy: one case of primary neural leprosy, one LL, and three BT cases. The reasons for temporary unfitness among these individuals included hepatitis B or C, Chagas disease, a positive VDRL test, or lipemic serum.

Among the five diagnosed cases, 2/5 (40.0%) tested positive by SSS bacilloscopy, 3/4 (75.0%) were positive by qPCR of SSS samples, and 2/5 (40.0%) were positive by qPCR of peripheral blood samples (Table 1).

**TABLE 1:** Detection of *Mycobacterium leprae* in slit skin smear samples and blood of temporarily unfit blood donors.

	Blood donors examined for leprosy	Unfit reasons	Bacillary index (slit skin smear)	qPCR‐RLEP (slit skin sample)	Ct (slit skin sample)	qPCR‐RLEP (blood)	Ct (blood)
Leprosy case	1	HCV	Negative	Negative	—	Negative	—
	2	Chagas disease	0.25	ND	—	Negative	—
	3	VDRL	5.0	Positive	32	Positive	39.71
	4	Lipemic serum	Negative	Positive	32.18	Negative	—
	5	HBV and HCV	Negative	Positive	40.95	Positive	42.19
Non‐leprosy case	6	HCV	Negative	Negative	—	Negative	—
	7	Lipemic serum	Negative	Negative	—	Negative	—
	8	VDRL	Negative	Negative	—	Negative	—

*Note*: The bacillary index and quantitative PCR (qPCR) RLEP results were categorised according to clinical examination status and the type of sample used for analysis.

Abbreviations: Ct, cycle threshold; ND, not done for technical reason related to the collected sample.

## DISCUSSION

Few studies have explored the possibility of leprosy transmission beyond the respiratory route. However, existing evidence suggests that transmission via the bloodstream may occur under specific circumstances, including accidental inter‐human exposure and experimental models in which blood cells from LL or borderline patients were inoculated into mouse footpads, resulting in infection [4].

In leprosy‐endemic regions of the Amazon, seroprevalence studies have shown that 29%–56% of schoolchildren test positive for anti‐PGL‐I IgM antibodies, indicating high levels of bacillary circulation in the population [8]. In our study, statistically significant differences in median OD values were observed between the seronegative group (0.146), the seropositive group (0.407), and the high‐titre group (1.023). These findings support the utility of anti‐PGL‐I IgM serology in distinguishing individuals with varying degrees of exposure to *M. leprae*, and potentially, different levels of transmission risk and disease progression.

Our results are consistent with two previous studies investigating leprosy in blood donors. One study reported 3.1% of donors as strongly seropositive and 19.4% as seropositive, although no new leprosy cases were identified [9]. Another study found 3.8% seropositivity, with a 0.6% incidence of new leprosy cases over a five‐year follow‐up period [10]. In our study, 5 out of 500 individuals (1.0%) were newly diagnosed with leprosy, a notably high prevalence considering the random sampling of temporarily unfit blood donors. These findings underscore the importance of immediate clinical evaluation using all available diagnostic tools—including physical examination, serology, qPCR, and ultrasound—followed by long‐term follow‐up.

Only the latter of the two referenced studies employed qPCR for all samples, detecting *M. leprae* DNA in 0.3% of blood samples. In contrast, our study focused on individuals with high anti‐PGL‐I IgM titres, inviting them for neurodermatological assessment. Of the 20 individuals with high titres, 8 (40.0%) agreed to clinical evaluation, and 5 (62.5%) were newly diagnosed with multibacillary leprosy. The remaining three individuals are under observation due to inconclusive clinical findings. Among the eight blood samples tested by qPCR, two (25.0%) were positive for the RLEP sequence. If extrapolated to the entire cohort of 500 donors, this would represent a 0.4% positivity rate, comparable to previous findings.

Our results confirm the high prevalence of anti‐PGL‐I IgM antibodies in the Amazon region [2], with a substantial proportion of individuals meeting clinical and laboratory criteria for leprosy diagnosis. Based on these findings, we recommend the following: routine anti‐PGL‐I IgM screening of blood donors in leprosy‐endemic regions to identify individuals at risk of subclinical or early‐stage disease; comprehensive clinical evaluation (including qPCR and ultrasound) for individuals with high anti‐PGL‐I IgM titres, followed by appropriate treatment and contact tracing; Surveillance studies to assess the potential for *M. leprae* transmission through blood donation and to inform transfusion safety policies in endemic and emerging areas.

## LIMITATIONS

We recognise some limitations that deserve consideration. The anti‐PGL‐I IgM cutoff adopted (OD ≥0.750) was based on prior experience and aimed to prioritise individuals with the highest likelihood of clinical disease; however, broader validation in population‐based cohorts is still needed. The follow‐up rate among high‐titre individuals was modest, reflecting challenges inherent to recontacting blood donors for clinical evaluation. We also acknowledge that comparisons with other case‐finding strategies—such as contact tracing—could provide valuable insights into the feasibility and impact of this approach. These aspects are being considered in the design of future studies.

## FUNDING INFORMATION

This work was supported by research grants from CAPES PROAMAZONIA 3288/2013, PROPESP/UFPA, CAPES, CNPq 313633/2018‐5 fellowship for CGS, and the Fulbright Scholar to Brazil for JSS. The study design, data collection, analysis, interpretation, and report writing were conducted independently of any involvement from the funders.

## CONFLICT OF INTEREST STATEMENT

The authors declare no conflicts of interest.

## ETHICS STATEMENT

The study was approved by the Research Ethics Committee of the Institute of Health Sciences, Federal University of Pará (CAAE 34990920.5.0000.0018 CEP‐ICS/UFPA), and participants provided written consent.
